# *Helicobacter pylori* infection in women with Hashimoto thyroiditis: A case-control study

**DOI:** 10.1097/MD.0000000000004074

**Published:** 2016-07-22

**Authors:** Haim Shmuely, Ilan Shimon, Limor Azulay Gitter

**Affiliations:** a Department of Internal Medicine D, Helicobacter Institute, Kaplan Medical Center, Rehovot and the Faculty of Medicine, Hebrew University, Jerusalem, Rehovot, Israel; b Institute of Endocrinology, Rabin Medical Center, Beilinson Hospital, Petach Tikva, Israel.

**Keywords:** cytotoxin-associated gene A, family history of thyroid disease, Hashimoto thyroiditis, *Helicobacter pylori*, thyroid malfunction

## Abstract

An association between *Helicobacter pylori* (*H pylori*) infection as environmental risk factors for Hashimoto thyroiditis (HT) has been reported. We investigated this hypothesis in women in which HT is more common. Serum immunoglobulin G antibodies against *H pylori* (enzyme-linked immunosorbent assay), CagA protein (Western blot assay), circulating antibodies to thyroid antigens, mainly thyroperoxidase (TPOAbs) and thyroglobulin (TgAbs), were tested in 101 females with HT and 111 non-HT control women without a history of autoimmune disease. Thyroid function, socioeconomic status at childhood, and family history of thyroid malfunction were also studied. Forty-seven HT women (46.5%) tested seropositive for *H pylori* versus 48 controls (43.2%; *P* = 0.63). The prevalence of anti-CagA antibodies was 21.3% in HT-infected patients and 31.2% in infected controls (*P* = 0.352). Women with HT were older than the controls at a significance level of 0.03, and higher prevalence of hypothyroidism (69% vs 13.5%, respectively) and family history of thyroid malfunction (59% vs 34%, respectively) (*P* < 0.001 in both). Body mass index, diaphragmatic hernia, peptic ulcer, heartburn, use of proton pump inhibitors, childhood socioeconomic background, and crowding index showed no significant difference between HT-positive or negative individuals. Multivariate analysis demonstrated that *H pylori* seropositivity was not associated with HT (odds ratio 1.15, 95% confidence interval 0.57–1.83, *P* = 0.95) and that family thyroid malfunction was independently associated with an increased risk of HT (odds ratio 3.39, 95% confidence interval 1.86–6.18, *P* < 0.001). No association was found between *H pylori* infection and HT in women. Family history of thyroid malfunction is a risk factor for HT.

## 1 Introduction

Hashimoto thyroiditis (HT), described over a century ago, is a chronic inflammation of the thyroid gland with undefined etiopathogenesis. HT is considered the most widespread autoimmune endocrine disorder^[1]^ and the most common cause of hypothyroidism.^[2]^

The classic form of HT typically presents during the fifth decade of life and is overwhelmingly more common in women (approximately a female-to-male ratio of 4:10).^[3]^ HT, diagnosed by a demonstration of circulating antibodies to thyroid antigens, mainly thyroperoxidase antibodies (TPOAbs) and thyroglobulin antibodies (TgAbs), remains a complex and ever expanding disease of unknown pathogenesis.

Infections caused by bacteria have been linked to autoimmune thyroid diseases (ATDs). *Helicobacter pylori* (*H pylori*) is the most common chronic bacterial infection affecting almost half of the world's population. Its most virulent strains identified by the presence of CagA antigens have been implicated in both organ specific and nonorgan specific autoimmune diseases.^[4]^

Several studies^[5–8]^ have shown a positive correlation between the presence of *H pylori* and HT; others have not.^[9,10]^ De Luis et al^[8]^ demonstrated that the titer of anti-*H pylori* immunoglobulin G (IgG) antibodies in Graves disease and HT was much higher compared with the controls. Yet, in a recent meta-analysis of 7 studies,^[11]^ which included 862 patients, it was reported that even though *H pylori* infection was associated with auto ATDs, the association was significant for Graves disease, and not for HT.

Our objective was to assess whether *H pylori* infection and CagA are associated with an increased risk for HT.

## 2 Methods

### 2.1 Setting

This case-control study was conducted at the Institute of Endocrinology, Rabin Medical Center, Beilinson Hospital—a 900-bed university-affiliated hospital, serving urban and nonurban populations of approximately 1 million as a first-line and tertiary facility.

### 2.2 Study design

Women aged 18 years or older were recruited from March 1 to August 31, 2013. Cases were consecutive women diagnosed with HT, referred to the Institute of Endocrinology. The control group, with no history of HT, was recruited via public advertisements from the same local community in central Israel. Subjects with hematological or solid malignancies, immunosuppression therapy, or other autoimmune diseases were excluded.

The study was reviewed and approved by the Institutional Review Board, Rabin Medical Center, Beilinson Hospital, Petach Tikvah, Israel. Informed consent was obtained from each patient. The study was partially supported by the Young Researcher's Grant, Rabin Medical Center, Beilinson Hospital, Petach Tikvah, Israel (Limor Azulai Giter).

Participants with a prior history of thyroid surgery, receiving radioactive iodine, cognitively impaired, unable to read, understand, or refused to sign the informed consent, were excluded from the study.

### 2.3 Variables

Diagnostic criteria of HT were positive serum titers of TPOAbs and TgAbs, anti-TPO >100 IU/mL, and anti-TG >150 IU/mL. Serum samples were tested for IgG antibodies against *H pylori* by an enzyme-linked immunosorbent assay (ELISA). The kit contains a partially purified protein preparation of *H pylori* collection strain NCTC 11637. The results were expressed as units per milliliter (U/mL) according to a calibrator curve. Values of ≥20 U/mL were considered seropositive, and values of <20 U/mL were considered seronegative for *H pylori*. Serum anti-CagA antibodies were analyzed using a CagA IgG kit ELISA. Levels of specific antibodies were expressed by units per milliliter (U/mL). Based on the presence of serum anti-CagA IgG (>6.25 U/mL), patients were classified as CagA-positive. Thyroid status (hyperthyroidism, hypothyroidism, euthyroidism) was evaluated by thyrotropin (TSH) (normal 0.3–4 mU/L), free thyroxine (FT4) (normal 10–20 pmol/L), and total triiodothyronine (TT3) (normal 1–2.8 nmol/L). Goiter prevalence was evaluated by palpation of the neck by a professional endocrinologist (IS).

Body mass index (BMI) was calculated. Comorbidities, current medication consumption, and family medical history were assessed by either an electronic medical record (EMR) or a medical interview. Subjects were interviewed using a validated structured questionnaire to determine childhood sociodemographic status. Queries included father's education, occupation, income, number of rooms, and number of people who lived in the house during childhood. Enrollees were also asked if they or any family members had a history of peptic ulcer diseases, gastric cancer, or thyroid malfunction. Age was classified as <30 years, 30 to <45 years, 45 to <60 years, or ≥60 years.

### 2.4 Data source/measurement

Blood samples (15 mL) were collected from each participant, centrifuged for 5 minutes and stored at −70°C until assayed. Serum anti-TPO and anti-TG antibodies were determined by ELISA using commercial kits (Orgentec).

Serum samples were tested for IgG antibodies against *H pylori* by ELISA using the Pyloriset EIA-GIII kit (Orion Diagnostica, Espoo, Finland) according to the manufacturer's instructions. The method, validated in our laboratory by a pilot study (data not shown), yielded a sensitivity of 94%, specificity of 90%, and positive and negative predictive values of 100% and 90%, respectively. Serum anti-CagA antibodies were analyzed using a CagA IgG kit (GD33; Genesis Diagnostics Ltd., London, UK), according to the manufacturer's instructions.

Thyroid function tests were performed by a chemiluminescent immunoassay (Immulite and Immulite 2000, Diagnostic Products Corp., Inc., Los Angeles, CA) used to measure TSH, FT4, and FT3.

Height and weight were measured by a trained nurse, and BMI was calculated. All subjects were interviewed by a trained staff member employing a validated structured questionnaire comprising demographic data, comorbidities, family medical history, and current drug consumption. Family history of hyper or hypothyroidism was defined as thyroid malfunction, because it is impossible to rely with absolute certainty that the report on the type of thyroid malfunction was accurate.

Childhood sociodemographic data included father's years of education and occupation (manual/nonmanual, other), father's income, crowding (number of siblings per room in the house), and the number of household members. All participants were examined by an endocrine and internal medicine specialist (IS).

### 2.5 Bias

To reduce bias, participants were informed that the information collected would not be used for any other purpose or affect their treatment. The questionnaire was also designed to reduce reporting bias. HT and *H pylori* criteria have both high sensitivity and specificity, and we therefore believe that classification biases were minimized.

Selection bias in the case group was minimized by using consecutive patients and a low rate of exclusions. The controls were offered no remuneration. We, therefore, believe that selection bias was minimal.

### 2.6 Study size

Prevalence of *H pylori* in the Israeli Jewish population (39%) was used as the expected prevalence in the control group. Figura et al^[6]^ reported an odds ratio (OR) of 3.78 between *H pylori* and HT. An OR of 2.5 was used to calculate the sample size. To evaluate the difference of a significant level of 5% and power of 80%, we needed 170 participants (85 in each group); ultimately, we were able to include a larger number of participants in each group which enabled a better statistical power.

### 2.7 Quantitative variables

To attain the highest statistical power, enrolled subjects were arbitrarily categorized into 4 age groups: <30 years, 30 to <45 years, 45 to <60 years, or ≥60 years to estimate age-specific *H pylori* prevalence. There is a reported increase of *H pylori* prevalence among these age groups in Israel with no significant difference between males and females.^[12,13]^

Malaty and Grahm^[14]^ found that low childhood socioeconomic class and high density of living increases the development of *H pylori* infection. For this reason, we included data as to the participants’ childhood socioeconomic status, that is, fathers with <8 years of education, working in unskilled occupations (manual workers), earning minimal wages versus fathers working in higher level occupations (clerk or manager), >8 years of education, and earning a higher wage.

Considering the epidemiological findings,^[15,16]^ we assessed household overcrowding at childhood, which may play a key role in the transmission of *H pylori* infection within the family. Crowding was defined as the number of people living in a household and the number of rooms.

### 2.8 Statistical analysis

Continuous variables were expressed as median and interquartile range (IQR). Categorical variables were summarized as frequencies and proportions. Continuous variables were compared using the Mann–Whitney *U* test. Differences in proportions among categorical data were assessed using the chi-square or Fisher exact test. The independent impact of *H pylori* on HT was assessed by multivariate analysis. Variables with a *P* value of <0.2 on univariate analysis were included in the multivariate analysis. Logistic regression was used for this purpose.

Odds ratios with a 95% confidence interval (CI) were reported. Statistical analysis was performed using the SPSS version 19. *P* value <0.05 was considered statistically significant. The statistical review of the study was carried out by an experienced epidemiologist.

## 3 Results

### 3.1 Patients

Of the 212 women recruited for this study, 101 were patients with HD and 111 were controls. None of the participants reported pregnancy. The demographic and socioeconomic characteristics of the study population and prevalence of *H pylori* and CagA antibodies are shown in Table 1.

**Table 1:**
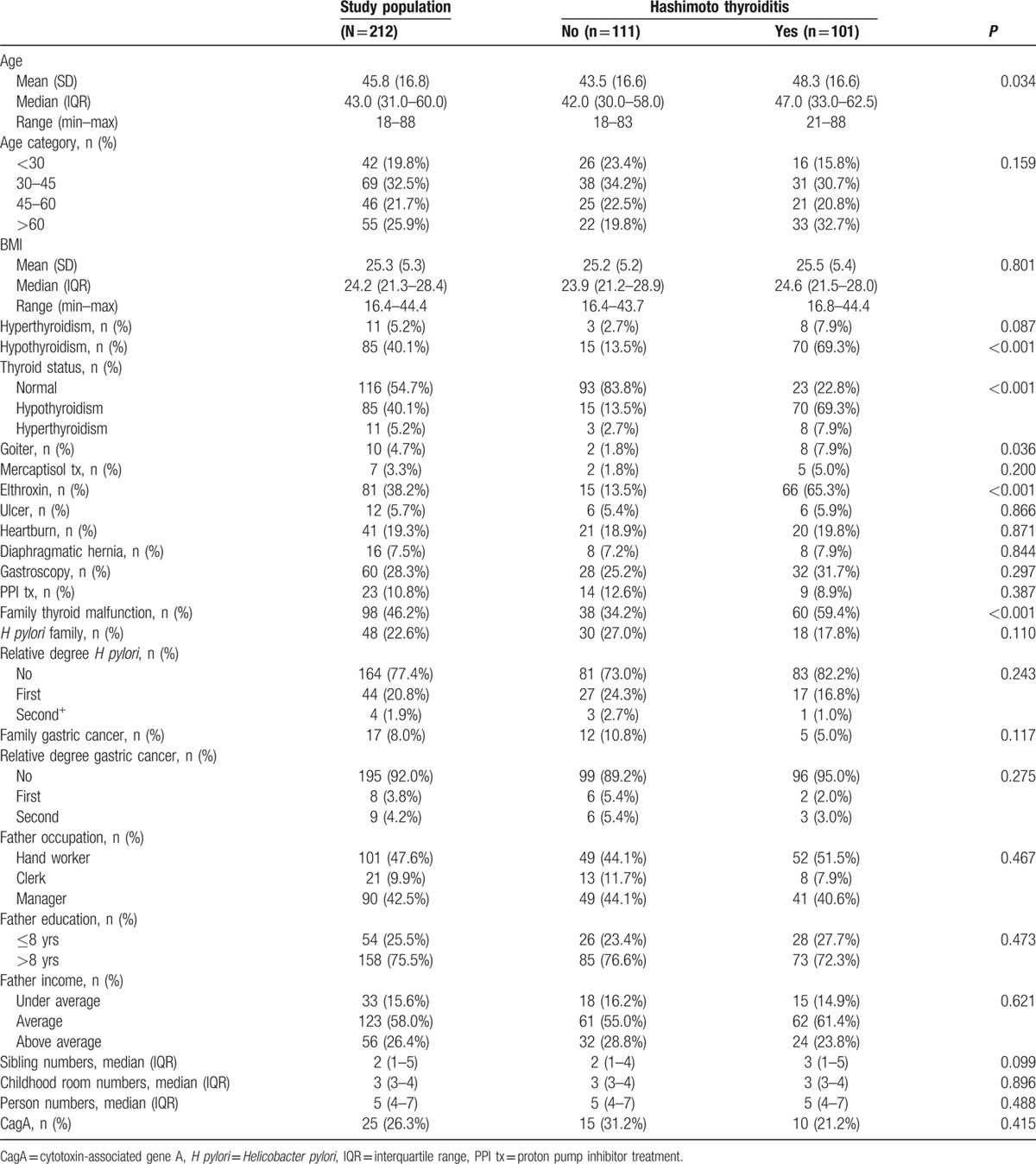
Demographic socioeconomic characteristics, BMI, thyroid status and treatment, family history, and gastrointestinal status of the study population and prevalence of *H pylori* and CagA antibodies.

Of 101 HT patients, 47 (46.5%) were positive for *H pylori* antibodies versus 48/111 (43.2%) controls (*P* = 0.63). CagA positivity in the serum of *H pylori*-positive subjects was found in 10/47 *H pylori*-positive (21.3%) HT patients; 15/48 (31.2%) *H pylori*-positive controls were positive for CagA antigens (*P* = 0.352). Once more, considering the overall prevalence of infection by CagA-positive *H pylori* in the studied groups (25/95, 26.3%), the results were not statistically significant (*P* = 0.41).

Women with HT were older than the controls at a significance level of 0.03, and higher prevalence of hypothyroidism (69% vs 13.5%, respectively) and family history of thyroid malfunction (59% vs 34%, respectively) (*P* < 0.001 in both). BMI, diaphragmatic hernia, peptic ulcer, heartburn, use of proton pump inhibitors (PPIs), childhood socioeconomic background, and crowding index showed no significant difference between HT-positive and negative individuals (Table 1).

Participants were further stratified into 4 age groups: <30 years, 30 to <45 years, 45 to <60 years, or ≥60 years old, elthroxine usage, and thyroid function (Table 2)*. H pylori* seropositivity, elthroxine usage, or thyroid function was not associated with HT in any of the age groups.

**Table 2:**
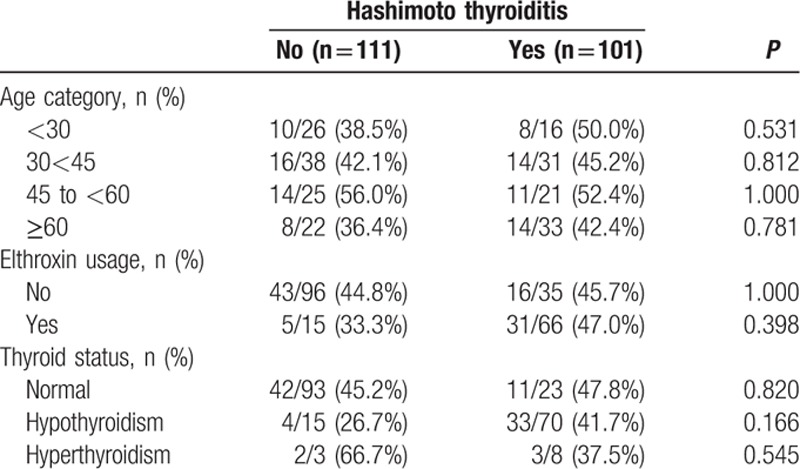
*Helicobacter pylori* prevalence in sub groups of patients with and without Hashimoto thyroiditis.

In the multivariate analysis, *H pylori* seropositivity was not associated with HT (OR 1.15, 95% CI 0.57–1.83, *P* = 0.95) (Table 3). A risk factor independently associated with an increased risk of HT was family thyroid malfunction (OR 3.39, 95% CI 1.86–6.18, *P* < 0.001).

**Table 3:**
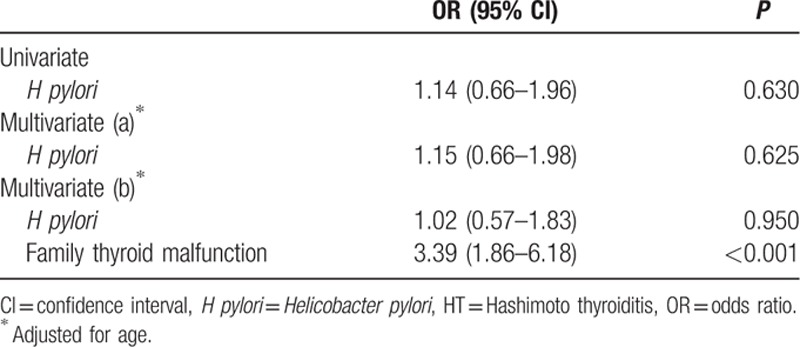
Multivariate model of risk factors independently associated with increased risk of HT.

## 4 Discussion

In this case-control study of women with HT versus non-HT women, an analysis of *H pylori* and CagA serum antibodies demonstrated that *H pylori* and CagA infection is not associated with HT in women. We have been unable to substantiate the association between HT and *H pylori* infection reported by several studies,^[5–8]^ because the acquisition of *H pylori* infection occurs primarily early in life, usually lasting for a lifetime if untreated.^[17]^ The socioeconomic status of the patients was assessed and analyzed to avoid bias when comparing cases and control groups. Our study had several limitations. Firstly, some of the controls had treated thyroid nonautoimmune malfunction. As such, these subjects may have had a different overall prevalence of thyroid disease compared with the general population. Secondly, a relatively high percentage of controls (13.5%) had hypothyroidism with negative antibodies. Perhaps, the cut-point for diagnosis was slightly higher, because Hashimoto thyroiditis is still the most common cause of hypothyroidism. In this study, goiter prevalence was evaluated by palpation of the neck by a professional endocrinologist. An additional thyroid ultrasound would have added data on the nodularity of the thyroid gland. Furthermore, our study did not include measurements of potential mediators of *H pylori*-related risks, such as gastrin levels, as a marker for chronic atrophic gastritis.^[18]^ Large prospective studies with healthy controls would be desirable.

Controls were older than the patient cases. *H pylori* infection is acquired during childhood,^[19]^ with almost no new infections appearing in adulthood.^[20]^ When comparing *H pylori* prevalence among the 4 age groups, no statistically significant association was found. We therefore believe that age was not associated with *H pylori* status.

Hashimoto thyroiditis is considered the most common autoimmune disease with a prevalence of 1/1000 persons per year.^[21]^ There is a very slight possibility that a participant in the control group could become an HT patient. Since a case-control study design was used, the influence of such an event would minimally affect the study results and would not change the study conclusion.

Our results were consistent with a recent meta-analysis^[11]^ demonstrating that *H pylori* infection is not associated with HT. The similar prevalence of *H pylori* infection (with and without CagA-positive strains) in women with *H pylori* and controls indicates that in contrast to reported observations,^[5–8]^ an association between *H pylori* infection and HT is unlikely. Some factors could be involved in the discrepancy between our results and previous data in the literature. De Luis et al^[8]^ studied only18 HD patients. Women primarily suffer from HT, which is the reason we included only women in our study; other studies have included both sexes.^[7,8]^ We studied only cases with HD, other studies included other ATDs in their analyses.^[6,8]^ Figura et al's^[6]^ study was partly designed retrospectively.

Whereas hypothyroidism is a characteristic functional abnormality, the inflammatory process early in the course of the disease may involve enough apoptosis to cause thyroid follicular disruption and thyroid hormone release, thus causing transient hyperthyroidism sometimes referred to as Hashitoxicosis.^[22]^

We evaluated the thyroid function in women with HT and the controls by measuring the serum levels of TSH and FT4, and found that *H pylori* seropositivity was not associated with thyroid function or elthroxine usage. We observed that HT in our patients was associated with a family history of thyroid malfunction. It is known that HT clusters in families occur either alone or in combination with Graves disease.^[23]^ A genetic susceptibility to HT^[24]^ with a sibling recurrence risk of >20^[25]^ has also been found.

In summary, the present study confirms that HT in women from central Israel had similar rates of serum *H pylori* antibodies compared with controls. A risk factor for HT included family history of thyroid malfunction. These results apply to this cohort only and may not be widely applicable. More studies performed on different populations are needed to confirm these findings.
